# Bicistronic Expression of a High-Performance Calcium Indicator and Opsin for All-Optical Stimulation and Imaging at Cellular Resolution

**DOI:** 10.1523/ENEURO.0378-22.2023

**Published:** 2023-03-28

**Authors:** Paul K. LaFosse, Zhishang Zhou, Nina G. Friedman, Yanting Deng, Anna J. Li, Bradley Akitake, Mark H. Histed

**Affiliations:** 1 Intramural Program, National Institute of Mental Health, National Institutes of Health, Bethesda, MD 20892; 2 National Institutes of Health-University of Maryland Graduate Partnerships Program, Bethesda, MD 20892; 3 Neuroscience and Cognitive Science Program, University of Maryland College Park, College Park, MD 20742; 4 Department of Biological Structure, University of Washington, Seattle, WA 98195

## Abstract

State-of-the-art all-optical systems promise unprecedented access to neural activity *in vivo*, using multiphoton optogenetics to allow simultaneous imaging and control of activity in selected neurons at cellular resolution. However, to achieve wide use of all-optical stimulation and imaging, simple strategies are needed to robustly and stably express opsins and indicators in the same cells. Here, we describe a bicistronic adeno-associated virus (AAV) that expresses both the fast and bright calcium indicator jGCaMP8s, and a soma-targeted (st) and two-photon-activatable opsin, ChrimsonR. With this method, stChrimsonR stimulation with two-photon holography in the visual cortex of mice drives robust spiking in targeted cells, and neural responses to visual sensory stimuli and spontaneous activity are strong and stable. Cells expressing this bicistronic construct show responses to both photostimulation and visual stimulation that are similar to responses measured from cells expressing the same opsin and indicator via separate viruses. This approach is a simple and robust way to prepare neurons *in vivo* for two-photon holography and imaging.

## Significance Statement

New multiphoton photostimulation methods, combined with standard two-photon calcium imaging, can yield unprecedented levels of control for dissecting brain circuit function *in vivo*. These all-optical methods rely on an interplay between optogenetics and calcium indicators, to both measure and control neural activity. However, genetic strategies to achieve reliable and stable co-expression of opsin and indicator are often challenging to execute. Here, we present a genetic tool to achieve robust co-expression of jGCaMP8s indicator and stChrimsonR opsin via a single injected virus. This approach facilitates all-optical experiments to investigate the circuit principles underlying brain activity.

## Introduction

Perception and action depend on neural computations, created as patterns of activity propagate in neural circuits. Means to monitor and control these activity patterns are important tools to study how brain function governs perception and behavior. Optogenetics is a valuable approach for controlling genetically-specified sets of neurons. However, achieving optical specificity, the ability to select a single cell and perturb it, is challenging. Conventional one-photon excitation is not ideal for single-cell stimulation in tissue, as one-photon excitation can lead to undesired activation above and below the targeted focal plane (Denk et al., 1990). Moreover, even in the focal plane, one-photon methods can be limited in their ability to restrict excitation to small volumes because of light scattering in the tissue (Denk et al., 1994), and this constraint becomes more severe with depth in the tissue.

Two-photon optogenetics overcomes these limitations, enabling perturbations in selected single cells (Rickgauer and Tank, 2009; Packer et al., 2012, 2015; Emiliani et al., 2015; Adesnik and Abdeladim, 2021). With this approach, using a stimulation laser and an imaging laser independently focused at different locations deep in the brain, it is possible to measure evoked activity patterns as stimulation is delivered. Two-photon optogenetics has been used to study within-area network dynamics (Chettih and Harvey, 2019), and to understand how chosen patterns of activity evoked by stimulation influence perception (Carrillo-Reid et al., 2019; Marshel et al., 2019; Dalgleish et al., 2020; Gill et al., 2020; Robinson et al., 2020; Daie et al., 2021; Rowland et al., 2021; Russell et al., 2022).

A variety of opsins and calcium indicators have been used for two-photon stimulation and simultaneous imaging (Shemesh et al., 2017; Mardinly et al., 2018; I.W. Chen et al., 2019; Marshel et al., 2019; Adesnik and Abdeladim, 2021; Forli et al., 2021; Sridharan et al., 2022). Desirable properties for calcium indicators used with two-photon stimulation include high sensitivity, to measure small changes in neural firing, and fast dynamics, to monitor quickly-changing spike trains. Desirable properties for opsins include fast dynamics to allow precise control of spiking, and the ability to be activated using moderate to low stimulation intensities. This allows many neurons to be stimulated with low total energy levels delivered to the brain.

However, expressing both an opsin and a calcium indicator in the same cells has proven challenging, especially to achieve stable expression levels of both proteins for weeks to months. High levels of indicator expression in single cells can lead to reduced fluorescence responses, typically with constant levels of bright fluorescence. This phenomenon of bright, nonresponsive neurons with high levels of calcium indicator expression can become more common as time elapses after transfection (Tian et al., 2009; T.W. Chen et al., 2013; Packer et al., 2015). Co-expression of the two proteins with two individual viruses allows fine-tuning and optimizing the expression level of each protein separately, but with this approach it can be challenging to achieve co-expression in many neurons (see Packer et al., 2015; Carrillo-Reid et al., 2018, their Fig. 4a; Chettih and Harvey, 2019; Gill et al., 2020; Russell et al., 2022). Genetic mouse lines promise to simplify this co-expression process (Bounds et al., 2022), but current genetic lines have limited combinations of opsin and calcium indicator available, and in genetic lines, it has not always been possible to achieve the levels of indicator expression (Daigle et al., 2018) that give imaging quality comparable to viral expression.

To address these issues, here we demonstrate a single Cre-dependent virus that expresses both opsin and indicator in transfected cells without requiring multiple overlapping viral injections. Our solution uses the ChrimsonR opsin, targeted to cells’ somata with a Kv2.1 domain (soma-targeted ChrimsonR, stChrimsonR; Pégard et al., 2017), and jGCaMP8s, a bright, sensitive genetically encoded calcium indicator (Zhang et al., 2021). The genes are linked by the self-cleaving peptide P2A (Szymczak et al., 2004; Prakash et al., 2012), an approach previously used with GCaMP6m and the opsin ChRmine (Marshel et al., 2019). stChrimsonR is an opsin with fast on- and off-kinetics (Klapoetke et al., 2014) with a red-shifted excitation spectrum (Sridharan et al., 2022) and moderate sensitivity to two-photon activation (Mardinly et al., 2018; I.W. Chen et al., 2019). This moderate sensitivity to stimulation and red-shifted excitation spectrum gives the advantage of allowing neurons to be driven by the stimulation laser, while reducing potential activation, or crosstalk, from the (lower peak intensity and blue-shifted) imaging laser. jGCaMP8s is from the latest generation of fast calcium indicators, balancing needs for a bright signal and physiologically relevant kinetics. We find this construct provides a stable preparation for long-term experiments with repeated stimulation. With this single virus strategy, we achieve widespread and stable expression, effective and precise holographic stimulation of many cells, and high-quality recording of neural activity.

## Materials and Methods

### Virus

Both pAAV-hSyn-DIO-ChrimsonR-mRuby2-ST (Addgene Plasmid #105448, RRID:Addgene_105448) and pGP-AAV-syn-jGCaMP8s-WPRE (Addgene Plasmid #162374, RRID:Addgene_162374) plasmids were used to build the pAAV-hSyn-DIO-jGCaMP8s-P2A-stChrimsonR construct and also used to make viruses expressing each protein individually. The mRuby fluorescent tag from pAAV-hSyn-DIO-ChrimsonR-mRuby2-ST was removed, and the sequence encoding jGCaMP8s was cloned into the construct along with a P2A peptide linker. The plasmid was used for packaging into an adeno-associated virus (AAV9). The plasmid version of this construct is available on Addgene (Plasmid #174007, RRID:Addgene_174007).

### Animals and surgery

All animal procedures were performed in accordance with NIH Institutional Animal Care and Use Committee (IACUC) regulations. *Emx1-Cre* mice (The Jackson Laboratory; RRID:IMSR_JAX:005628) were used in all experiments to target expression of Cre to excitatory, glutamatergic neurons. *N* = 8 total animals were used in this study (*N* = 4 male, *N* = 4 female); no differences because of sex were noted in the results. Mice two months of age or older were anesthetized with isoflurane (1–3% in 100% O_2_ at 1 l/min) and kept on a heating pad for warmth. An intraperitoneal injection of dexamethasone (3.2 mg/kg) was administered before incision to reduce inflammation. The skull was exposed, and a custom metal head post was positioned at the base of the skull. A 3-mm diameter circular craniotomy was made over the left hemisphere of primary visual cortex (V1; ML −3.1 mm, AP +1.5 mm relative to λ) using an air driven dental drill (Aseptico) with a Neoburr drill bit (Friction Grip 1/4; Microcopy). AAV9-hSyn-DIO-jGCaMP8s-P2A-stChrimsonR was diluted in PBS, and 2 nmol sulforhodamine 101 was added to visualize injection progression in the brain. We tested a small range of viral titers (2.6–4.7 × 10^12^ GC/ml) in six animals and found all these titers allowed for reliable data collection (titers: 2.6 × 10^12^ GC/ml in mice 1, 3, 4, and 6; 3.4 × 10^12^ in mouse 2; 4.7 × 10^12^ in mouse 6).

For “dual-virus”-injected animals, we instead diluted in PBS two separate viruses: AAV9-hSyn-jGCaMP8s-WPRE (final titer 1.0 ×10^13^ GC/ml) and AAV9-hSyn-DIO-stChrimsonR-mRuby2 (final titer 2.7 × 10^12^ GC/ml).

Viruses were injected unilaterally with a stereotactic syringe pump (Stoelting) through a pulled glass pipette tip cut to an opening of 10- to 15-µm diameter. Injections were targeted to 200 µm below the surface of the brain and administered at a rate of 0.1 µl/min for a total volume of 300 nl per injection site (5–10 injection sites). A 3-mm optical window (Tower Optical) was implanted over the craniotomy. Both the optical window and metal head post were fixed to the skull using C&B Metabond dental cement dyed black (Parkell). Last, a custom-made removable light-blocking cover was fixed atop the implant to prevent ambient light exposure to the opsin. Animals were individually housed after surgery. Mice were imaged three or more weeks postinjection. All animals were housed in a 12/12 h reverse light/dark cycle and allowed food and water *ad libitum*.

### Widefield fluorescence imaging

**Figure 1.**
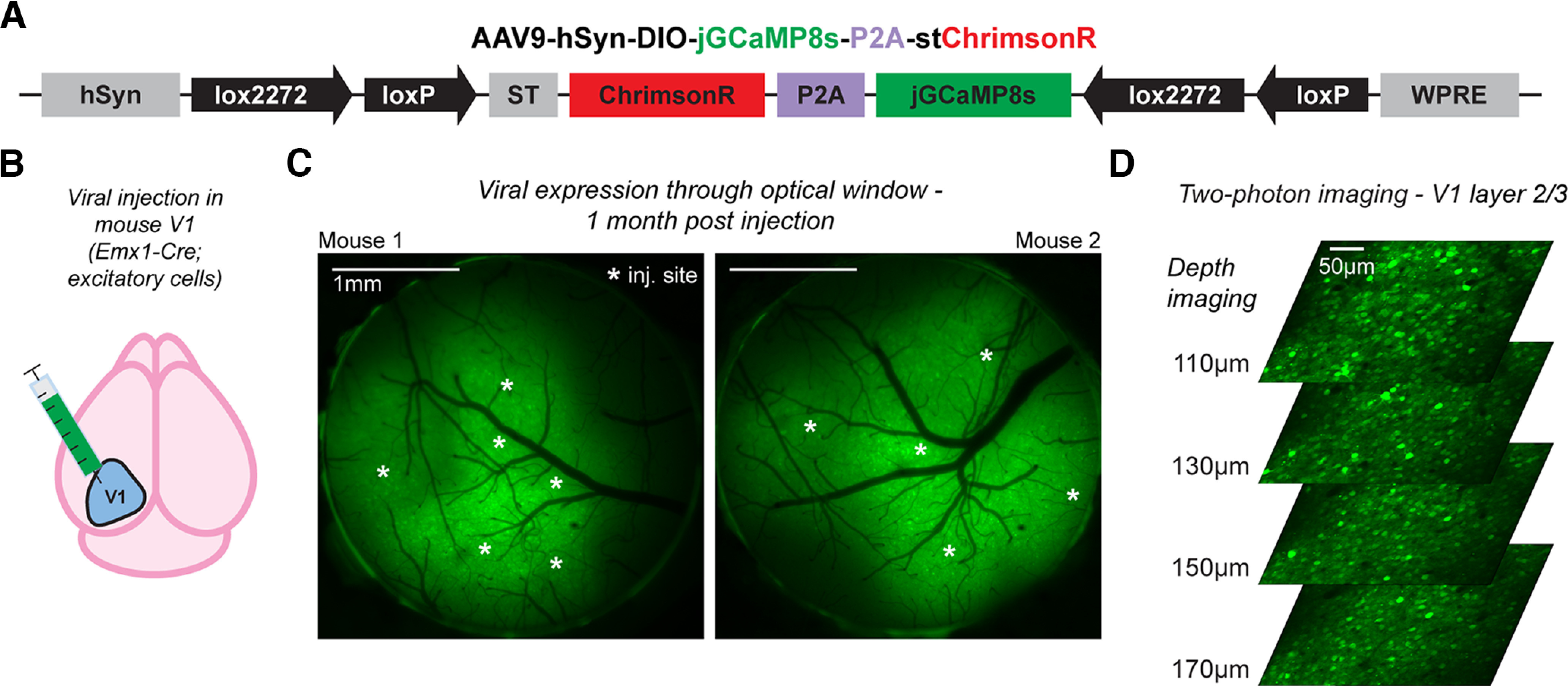
Widespread and stable co-expression of jGCaMP8s and stChrimsonR in mouse cortical neurons with a bicistronic viral vector. ***A***, Schematic of the Cre-dependent vector. ***B***, Viral injections were made in primary visual cortex (V1) to achieve targeted expression in excitatory neurons using an *Emx1-Cre* mouse line. ***C***, Widefield fluorescence imaging through 3-mm optical windows in two example mice shows widespread expression over the cortical surface surrounding injection sites (white asterisks); see also Extended Data Figure 1-1. ***D***, Two-photon calcium imaging (at 920 nm) in mouse V1 throughout layer 2/3 shows robust expression of the virus across many cells (FOV 414 × 414 µm); imaging produces minimal to no stimulation response (Extended Data Fig. 1-2). This figure: *N* = 3 mice. ***C***, Mice 1–2. ***D***, Mouse 3.

Widefield fluorescence imaging was done using a Discovery stereo microscope (Zeiss) with an X-Cite XYLIS LED source (Excelitas) and a blue excitation and green emission filter set (KSC XXX-814; Kramer Scientific). Images (Fig. 1, Extended Fig. 1-1) were collected (200-ms exposure period) using a Retiga R3 CCD camera (QImaging).

10.1523/ENEURO.0378-22.2023.f1-1 Extended Data Figure 1-1 Widefield fluorescence imaging of jGCaMP8s-P2A-stChrimsonR expression. Widefield fluorescence imaging through 3-mm optical windows in experimental mice. White stars: injection sites for each mouse (mouse 3: *N* = 5 sites, mouse 4: *N* = 10 sites, mouse 5: *N* = 10 sites, mouse 6: *N* = 6 sites). Images were acquired 22–53 d after injection. Download Figure 1-1, TIFF file.

**Figure 2.**
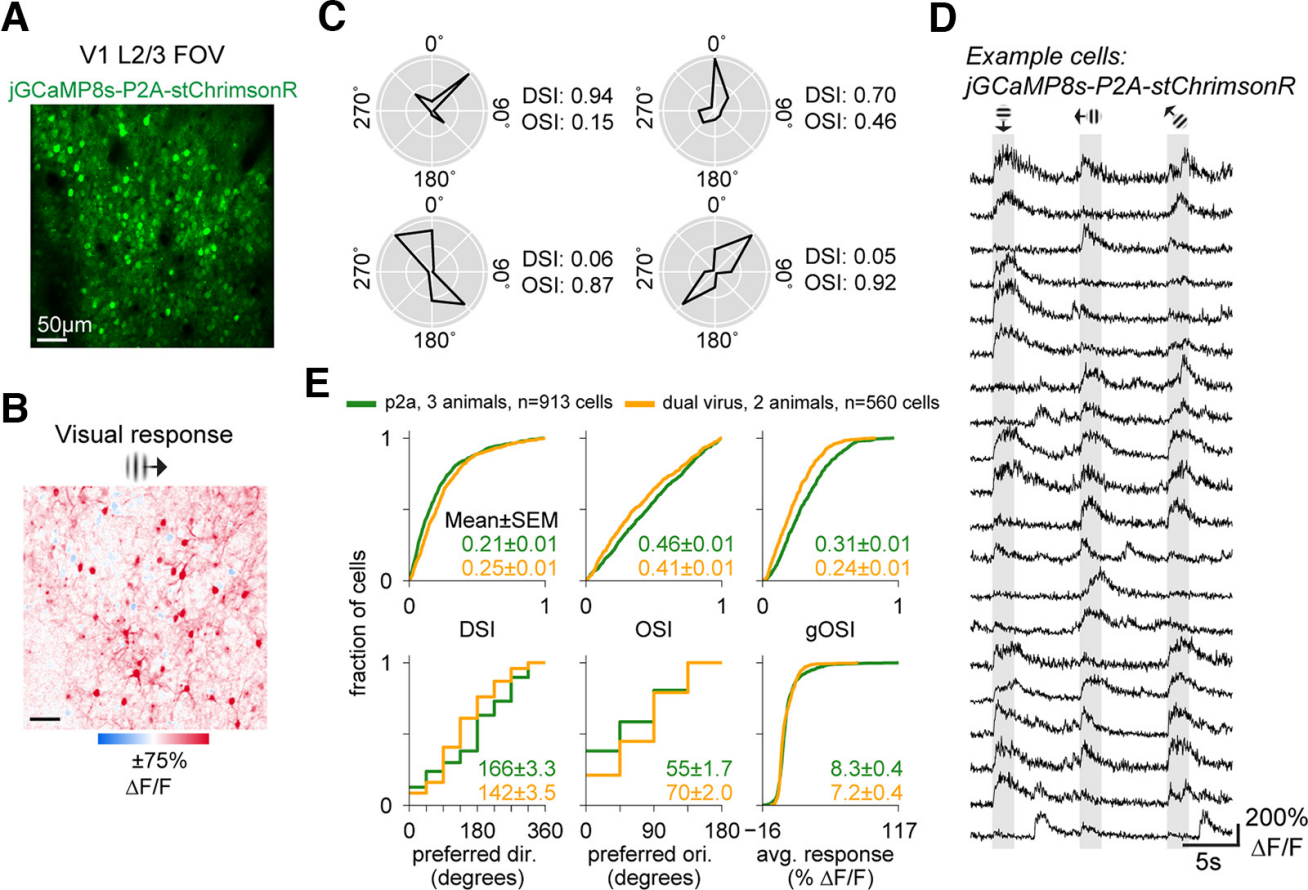
V1 cells expressing jGCaMP8s-P2A-stChrimsonR show expected visually-evoked and spontaneous activity. ***A***, Two-photon imaging FOV (414 × 414 µm) in layer 2/3 of mouse V1 expressing jGCaMP8s-P2A-stChrimsonR. Imaging: 30-Hz frame rate, 15 mW, 920 nm. ***B***, Example trial-average visual responses to a retinotopically-aligned 15° FWHM Gabor. One orientation shown. (In total, 8 orientations presented in random order, each 2-s duration, *N* = 20 repetitions/orientation.) ***C***, Trial-average responses in four example cells showing direction and orientation tuning. DSI and OSI calculated using average ΔF/F_0_ across the 2-s visual period. Polar plots, Responses normalized to the peak response across all eight orientations for each cell. ***D***, ΔF/F_0_ traces in example cells (*N* = 20), showing stimulus-evoked responses across three consecutive trials. ***E***, Cumulative distribution functions showing distributions of tuning metrics and average responsivity (average response across 8 directions) for all cells from P2A mice (*N* = 931 cells across 3 mice, green lines) and dual-virus mice (*N* = 560 cells across 2 mice, orange lines). DSI: direction selectivity index, OSI: orientation selectivity index, gOSI: global orientation selectivity index. This figure: *N* = 5 mice. ***A–D***, Mouse 4. ***E***, Mice 3–5, and two dual-virus mice.

10.1523/ENEURO.0378-22.2023.f1-2 Extended Data Figure 1-2 Minimal signs of cross talk activation from resonant-galvo scanning at imaging onset. Imaging cross talk should lead to elevated fluorescence at onset of imaging. Cell-averaged fluorescent traces in imaging FOVs (*N* = 12 FOVs from *N* = 5 mice) across first 2 s of imaging do not show signs of widespread cross talk in activation from the imaging laser (920 nm). Only a few sessions (2/12) show small increases in fluorescence over the first few seconds after imaging onset, as would be expected if imaging cross talk were occurring (significantly higher levels of fluorescence in the 2nd second of imaging vs the 1st second *p* < 0.05, Mann–Whitney *U* test with Bonferonni correction for multiple comparisons). On the other hand, however, four of 12 imaging sessions show significantly lower levels of fluorescence, arguing against cross talk activation (*p* < 0.05, Mann–Whitney *U* test with Bonferroni correction for multiple comparisons). Together, this suggests that elevated fluorescence at the start of imaging, due to cross talk, is not a concern with this preparation. Download Figure 1-2, TIFF file.

### *In vivo* two-photon calcium imaging

**Figure 3.**
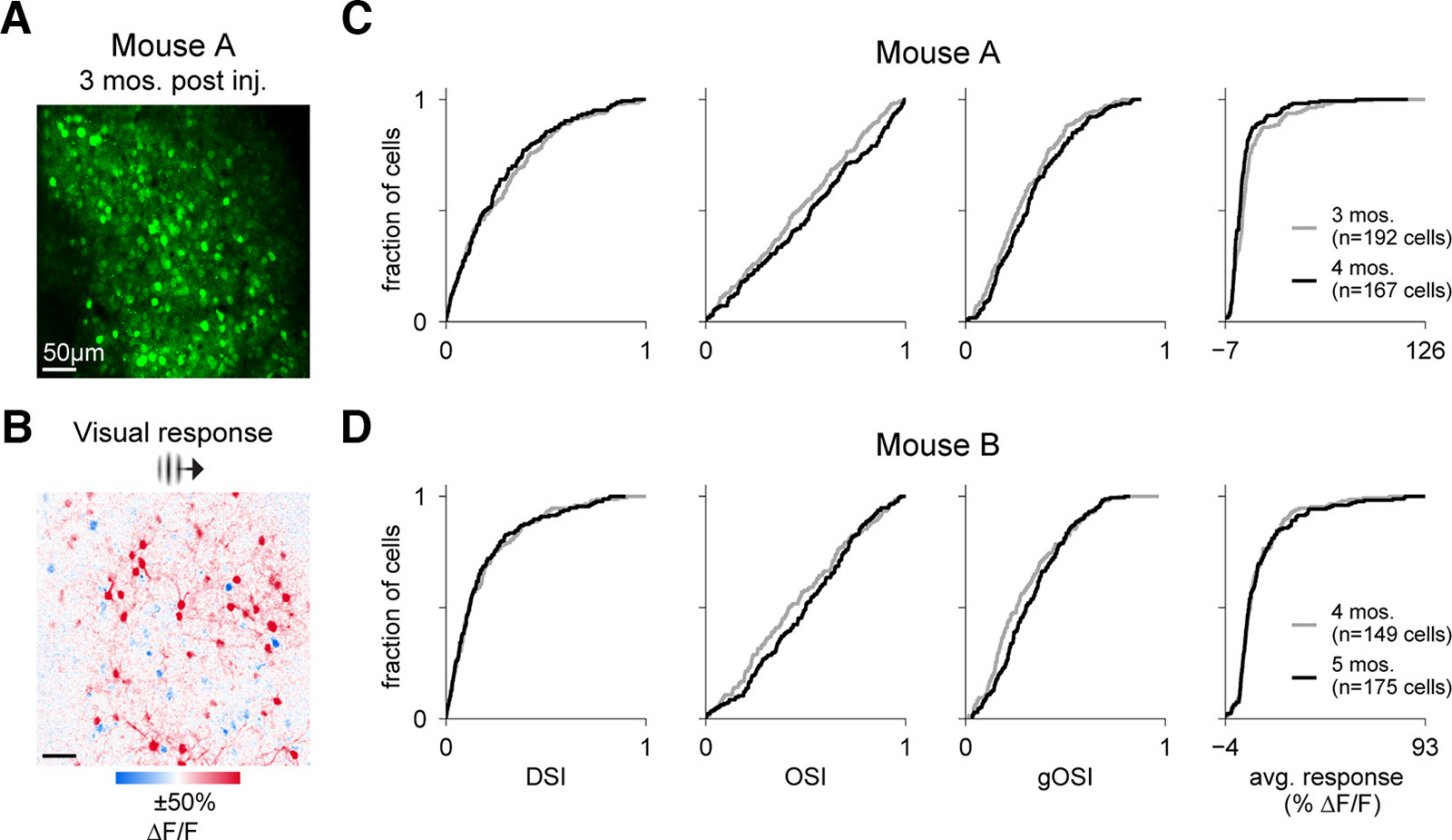
V1 cells expressing jGCaMP8s-P2A-stChrimsonR maintain visual responses for weeks following injection. ***A***, Two-photon imaging FOV (414 × 414 µm) in layer 2/3 of mouse V1. Imaging: 30-Hz frame rate, 15-mW power, 920 nm. ***B***, Example trial-average visual responses to a retinotopically-aligned Gabor (15° FWHM) showing evoked activity (ΔF/F_0_) for a single orientation. ***C***, Cumulative distribution functions showing distributions of tuning metrics and average responsivity (average response of all 8 directions) across all cells from the mouse in ***A*** at two time points a month apart in the same FOV (*N* = 192 cells at 3 months postinjection, gray line, *N* = 167 cells at 4 months, black line). ***D***, Same as in ***C***, but for a second mouse (*N* = 149 cells at 4 months postinjection, gray line, *N* = 175 cells at 5 months, black line). This figure: *N* = 2 mice. ***A****–****C***, Mouse 3. ***D***, Mouse 6.

**Figure 4.**
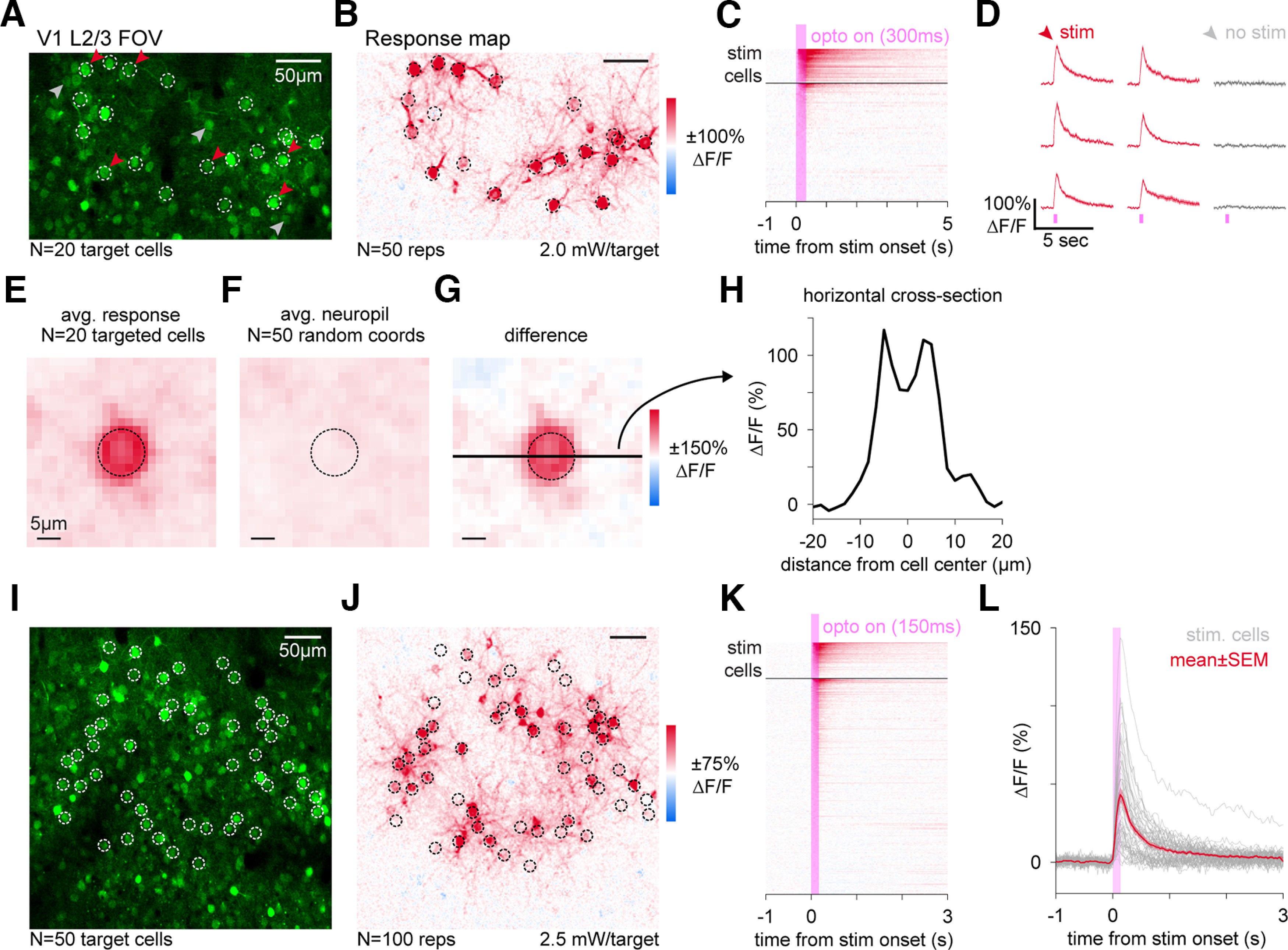
Simultaneous holographic stimulation and calcium imaging in cells expressing jGCaMP8s-P2A-stChrimsonR. ***A***, Two-photon imaging FOV (340 × 210 µm) in mouse V1, depth 122 µm from the pia. Imaging: 30-Hz frame rate, 10-mW power, 920 nm. White rings, Holographic stimulation locations (*N* = 20). Red, gray arrows, Examples of stimulated and nearby unstimulated cells, shown in panel ***D***. ***B***, Responses to stimulation (ΔF/F_0_) in same FOV as ***A*** (*N* = 50 stim repetitions, 2.0 mW/target, 300-ms stim duration, 10-µm diameter holographic disk pattern, stim. delivered during imaging acquisition and gated off at edges of scan field, reported powers are adjusted for fast-gated duty cycle; Materials and Methods). ***C***, Trial-average activity traces for all cells (*N* = 89 cells). Black horizontal bar separates stimulation targets (above), other cells in the FOV (below). ***D***, Trial-average cell traces (mean ± SEM, *N* = 50 repetitions) for nine example cells (*N* = 6 stimulated, red, and *N* = 3 unstimulated, gray). Error bars (SEM) are small enough to be visible only as slightly thickened lines. Decay comparison to dual-virus expression (Extended Data Fig. 4-1). Distributions of responses in stimulated and unstimulated cells, Extended Figure 4-2. ***E***, Average response map centered on targeted cells from ***A***, ***B***. ***F***, Average neuropil responses centered on 50 randomly selected coordinates. ***G***, Difference of maps from ***E***, ***F***. Horizontal black bar: cross-section in ***H***. Dashed circle: 10-µm diameter holographic disk pattern. ***H***, Horizontal cross-section of response map in ***G***. FWHM of response = 13.5 µm. ***I*–*K***, Same as ***A–C***, in a different FOV in the same mouse one month later (414 × 414 µm, depth = 130 µm, *N* = 50 targets, *N* = 348 total cells, *N* = 100 stim reps, 16-mW imaging power, stimulation power 2.5 mW/target, 150-ms stim duration, 10-µm diameter disk pattern). ***L***, Trial-average cell traces (*N* = 100 stim repetitions). Gray lines, 50 targeted cells in ***I***; red line, mean ± SEM cell trace. In *N* = 3 animals with 10–12 stimulated target cells: 30/32 targeted cells responsive, 35/1037 unstimulated cells responsive (see Extended Data Figs. 4-3, 4-4). *N* = 4 photostimulation animals, *N* = 1 this figure, mouse 1. Extended Data Figure 4-3: *N* = 3 mice, mice 3, 4, 6.

To perform two-photon calcium imaging, animals were first head-fixed under a 16× water-immersion objective (Nikon). Imaging was performed using a custom-built microscope using MIMMS (Modular In vivo Multiphoton Microscopy System) components (Sutter Instruments) and a Chameleon Discovery NX tunable femtosecond laser (Coherent). Imaging was controlled using ScanImage software (MBF Biosciences) in MATLAB. A small volume (∼1 ml) of clear ultrasound gel was placed over the optical window to immerse the objective. Calcium responses were measured at ∼100–200 µm below the surface of the pia in layers 2/3 of primary visual cortex (Figs. 2-4) using a 414 × 414 µm field of view (FOV), except where noted. Imaging was performed via bidirectional raster scanning with a resonant-galvo system (8-kHz resonant scanner, 512 lines, ∼30-Hz frame rate) using 920-nm wavelength light at 15–20 mW measured at the front aperture of the objective (pulse rate 80 MHz, pulse energy 0.19–0.25 nJ/pulse).

10.1523/ENEURO.0378-22.2023.f4-1 Extended Data Figure 4-1 GCaMP decay times to photostimulation responses are similar between P2A and dual-virus preparations. ***A***, Two-photon imaging FOV (414 × 414 µm) in layer 2/3 of mouse V1 for both a P2A mouse (top) and a dual-virus mouse (bottom). Imaging collected at 30-Hz frame rate, 10-mW power, 920 nm. ***B***, Trial-average photostimulation responses in five example cells from a P2A (left, green lines) or dual-virus (right, orange lines) mouse. Black lines, Exponential decay functions fit to the 600-ms period following stimulation offset for all photostimulated cells to estimate decay times (Materials and Methods). Stimulation power was 2.0 mW/target (P2A) or 2.5 mW/target (dual-virus) for 300 ms using 10-µm diameter disk patterns. ***C***, Half-decay times for photostimulated cells from both mice. Horizontal black lines, Mean half-decay time amongst cells. Vertical black lines, SEM. Half-decay times are not significantly different between P2A-expressing and dual-virus-expressing cells (*p* = 0.37, two-sample *t* test). This figure: *N* = 2 mice. ***A–C***, Mouse 1 and a dual-virus mouse. Download Figure 4-1, TIFF file.

10.1523/ENEURO.0378-22.2023.f4-2 Extended Data Figure 4-2 Distribution of responses to photostimulation in stimulated and unstimulated populations. ***A***, Cumulative distribution functions of photostimulation responses (from data in Fig. 4*A–D*) averaged over a 1-s period following stimulus onset in both stimulated cells (red) and unstimulated cells in the FOV (black). Using a 7.5% ΔF/F_0_ threshold, we found 19/20 stimulated cells and 5/69 unstimulated cells show reliable activation. ***B***, Same as in ***A***, but for data from Figure 4I-L. Using a 7.5% ΔF/F_0_ threshold, we found 33/50 stimulated cells and 45/298 unstimulated cells show reliable activation. To account for concerns of stronger neuropil contamination in response calculations when stimulating larger groups of cells, we also used a ring-based neuropil correction via Suite2p and found minimal differences in responses (35/50 stimulated cells and 41/298 unstimulated cells above a 7.5% ΔF/F_0_ threshold), implying neuropil signal was not contaminating cell responses. This figure: *N* = 1 mouse. ***A***, ***B***, Mouse 1. Download Figure 4-2, TIFF file.

10.1523/ENEURO.0378-22.2023.f4-3 Extended Data Figure 4-3 Holographic stimulation of cells expressing jGCaMP8s-P2A-stChrimsonR. ***A***, Two-photon imaging FOV (414 × 414 µm) in mouse V1 in three example mice. [Depths: 130 µm (top), 140 µm (middle), and 110 µm (bottom); each in L2/3, depth differences do not produce systematically different responses.] Imaging at 30-Hz frame rate, 15-mW imaging power, 920 nm. White dashed rings, Holographic stimulation pattern (top: *N* = 10 targets, middle: *N* = 12 targets, bottom: *N* = 10 targets). ***B***, Corresponding stimulation response maps showing mean optogenetically-evoked activity (ΔF/F_0_; *N* = 50 stim reps). Stimulation power 2.5 mW/target, stim duration 300 ms, 10-µm diameter disk patterns. ***C***, Trial-average activity of all stimulated cells (light gray lines) plotted with mean ± SEM activity across all stimulated cells (red lines) and all unstimulated cells (black lines). SEM is present for unstimulated cells, but small relative to the size of the plotted line. Fraction of responsive stimulated cells: (top) 9/10, (middle) 11/12, (bottom) 10/10 (using 7.5% ΔF/F_0_ threshold, see Materials and Methods for details). Fraction of responsive unstimulated cells: (top) 11/436, (middle) 19/430, (bottom) 5/171. Download Figure 4-3, TIFF file.

10.1523/ENEURO.0378-22.2023.f4-4 Extended Data Figure 4-4 Spatial extent of photostimulation effects in unstimulated cells. Trial-average responses to photostimulation. Red points, Stimulated cells. Gray points, Unstimulated cells. Unstimulated cells are plotted as a function of distance to the nearest stimulated cell in the FOV. Stimulation power, duration, and the mean pairwise distance between stimulated targets is shown for all photostimulation experiments in P2A animals. This figure: *N* = 4 mice. Download Figure 4-4, TIFF file.

### *In vivo* two-photon holographic photostimulation

Holographic photostimulation was performed using a Satsuma femtosecond pulsed laser (Amplitude Laser) at 1030-nm wavelength along a second optical path (galvo-galvo) integrated into the two-photon microscope just before the tube lens using a polarizing beam combiner. A spatial light modulator, or SLM (1920 × 1152 pixels; Meadowlark Optics), was used to generate holographic patterns of 10-µm diameter disks within a two-dimensional plane (aligned to the imaging focal plane). The SLM was followed by a relay lens system (two achromatic lenses, with focal lengths 250 mm and 100 mm) in a 4-f configuration between SLM and galvanometers. Galvonometers were used to direct the diffracted light pattern from the SLM onto the sample, pointing the center of the diffracted pattern to the center of the targeted cells to maximize diffraction efficiency. The zero order (undiffracted beam) from the SLM was blocked using a small amount of furnace cement (30- to 40-µm diameter) on a glass slide. Stimulation targets were defined and SLM phase masks were computed using ScanImage software (MBF Biosciences). The radial point-spread function (PSF) of diffraction limited spots generated by the SLM was 0.73 µm and the axial PSF was 9.85 µm.

Stimulation was applied for intervals of either 150 ms at 2 mW/target (Fig. 4*A–H*) or 300 ms at 2.5 mW/target (Fig. 4*I–L*) with a 500-kHz pulse rate (pulse energy 13 or 16 nJ/pulse). The laser was gated on when the imaging resonant galvo was reversing direction during bidirectional scanning, and off during the imaging pixel acquisition (on time 19 µs, off time 44 µs, duty cycle 30%). This fast gating allows for imaging neural responses during stimulation periods, by collecting imaging data on each line while stimulating in between lines, switching at rates (period 62.5 µs) much faster than the onset kinetics of the opsin (>1 ms; Klapoetke et al., 2014; Mardinly et al., 2018; Sridharan et al., 2022). To perform the fast gating, the imaging frame clock was inverted via a TTL logic gate (Pulse Research Lab; see Mardinly et al., 2018 for example circuit). We report average stimulation power over the milliseconds-long stimulation pulses: that is, the average power we report is found by multiplying the peak stimulation power while the laser is on during the fast pulses by the duty cycle of the fast pulses. For example, for Figure 4*A–H*, the average power per target was 2 mW (the number we report), while the peak power of the laser over the target was 6.5 mW and the duty cycle 30%.

### Retinotopic mapping

To map the retinotopic position of visual stimuli in V1 under the optical window, we performed hemodynamic intrinsic imaging in awake head-fixed animals. We presented visual stimuli (drifting square wave gratings, 0.1 cycles/°, 10° diameter) for 5 s (with 10 s between presentations) at different retinotopic positions and measured reflected 530-nm light on the brain to quantify hemodynamic-related changes in absorption. The 530-nm light was delivered using a fiber-coupled LED (M530F2; Thorlabs) and imaging was collected on the same stereo microscope used for widefield fluorescence imaging using a 1× widefield objective and a green long-pass emission filter. Imaging data was acquired at 2 Hz. Changes in reflectance were computed for every stimulus location between a baseline period (5 s before stimulus onset) and a response period (2.5-s window starting 3 s after stimulus onset). A centroid of the hemodynamic response was computed for each stimulus location and an average retinotopic map was fit to the positions of the centroids. Retinotopic maps were then used to guide stimulus locations for two-photon imaging measures of visual responses to Gabor stimuli.

### Visual stimulation

To measure visual responses during two-photon imaging, we presented awake animals with Gabor patches [sinusoidal drifting gratings filtered with a Gaussian mask with 15° full-width half-max (FWHM)] with spatial frequency 0.1 cycles/° for 2-s periods (6 s of gray screen between presentations) at 100% contrast. An LCD monitor with neutral gray background was used to present visual stimuli and was positioned ∼20 cm in front of the animal. Drifting gratings of eight different directions (45° increments) were presented in random order across trials. Each direction was presented for 20 repetitions.

### Two-photon calcium imaging analysis

Two-photon calcium imaging data were first downsampled from 512 × 512 to 256 × 256 pixels to ease handling of data. Background correction was performed by computing the average intensity image across frames and subtracting the minimum pixel value of this average from the image stack. All remaining negative pixel values (because of noise) were then set to zero. We motion corrected all images using the CaImAn toolbox (Giovannucci et al., 2019) and performed cell segmentation using Suite2p to allow manual selection of cell masks (Pachitariu et al., 2017). Fluorescence intensity traces were then calculated as the average intensity across all pixels within a cell’s segmented mask. To quantify cell activity, we computed ΔF/F_0_ for each cell. F_0_ was defined as the average fluorescence across the 50 imaging frames that occurred directly before stimulus presentation for all trials (*N* = 160 trials, Figs. 2, 3; *N* = 50 trials, Fig. 4*C*; *N* = 100 trials, Fig. 4*K*) in an imaging session.

Reliability measures for visual responses were quantified via two-sample *t* test (with Bonferroni correction for multiple comparisons within each experiment) using ΔF/F_0_ values between the visual stimulation period (*N* = 60 frames) and an equivalent number of frames preceding stimulus onset across all trials. Photostimulation responses were determined by averaging ΔF/F_0_ activity over a 1 s period (*N* = 30 frames) following stimulus onset and assessed using a 7.5% ΔF/F_0_ threshold (Extended Data Fig. 4-2).

For visual display of ΔF/F_0_ responses across the field of view (FOV; in Figs. 2*B*, 3*B*, 4*B*; Extended Data Fig. 4-3*B*), F_0_ was computed for every pixel in the same manner as for cell-based calculations. We then smoothed the F_0_ image using a Gaussian filter (σ = 20, radius = 80.5 pixels) to act as a means of local contrast adaptation. F was computed at every pixel as the average value across a response window [*N* = 60 frames (Figs. 2*B*, 3*B*), *N* = 10 frames (Fig. 4*B*; Extended Data Fig. 4-2*B*); *N* = 15 frames (Fig. 4)] following stimulation onset and across all trials (for Figs. 2*B*, 3*B*, *N* = 20 trials/stimulus direction).

To assess the lateral extent of photostimulation, we quantified the average activity across 40 × 40-µm regions centered on targeted cells (from Fig. 4*B*). To control for activation of surrounding neuropil across the entire field of view, we subtracted an average neuropil region of interest (ROI) centered on 50 random coordinates in the field of view (Fig. 4*F*,*G*). A Gaussian specified by:

$$y\;=\;ae^{-{\left(x-b\right)}^{2}/2c^{2}}$$

was fit to the one-dimensional cross-section of the average cell-centered response ROI (Fig. 4*G*). The lateral extent of a photostimulation disk target (10 µm) was found as the full width at half-max of this Gaussian fit.

### Half-decay time of photostimulation responses

To assess the decay time of photostimulation responses, stimulus-triggered average responses were computed for each cell. Exponential decay functions of the form:

$$y\;=\;ae^{-t/\tau }$$

were fitted to the 600-ms (20 frames at 30 Hz) period immediately after photostimulation offset. The half-decay time was calculated as:

ln(2)τ

### Visual tuning analysis

To quantify visual tuning in individual cells, we computed a direction selectivity index (DSI), orientation selectivity index (OSI), and global orientation selectivity index (gOSI) for all visually-responsive cells following the methods of Kondo and Ohki (2016). For each metric, 0 is minimum and 1 is maximum selectivity. We first calculated a tuning curve for each cell as the average ΔF/F_0_ activity across the entire visual stimulus period (*N* = 60 frames, or 2 s) and across all trials for each of the eight stimulus directions (*N* = 20 trials/direction). Responsivity was calculated as the average value across the eight directions in the tuning curve. The stimulus direction corresponding to the peak value of this tuning curve was the preferred direction for a cell. The response at this direction, R_prefDir_, as well as the opposite direction (180° away), R_oppoDir_, was used to calculate the DSI as:

$$\text{DSI }=\;\left({\text{R}}_{\text{prefDir}}-{\text{ R}}_{\text{oppoDir}}\right)\;/\;\left({\text{R}}_{\text{prefDir}}+{\text{ R}}_{\text{oppoDir}}\right)$$

To calculate OSI, we first averaged opposite pairs of directions of the tuning curve to yield average responses to each of the four stimulus orientations. The preferred orientation of cells was determined to be the peak value between the four orientations. The response at the preferred orientation, R_prefOri_, as well as the response at the orthogonal orientation (90° away), R_orthOri_, was used to calculate the OSI:

$$\text{OSI }=\;\left({\text{R}}_{\text{prefOri}}-{\text{ R}}_{\text{orthOri}}\right)\;/\;\left({\text{R}}_{\text{prefOri}}+{\text{ R}}_{\text{orthOri}}\right)$$

Last, the gOSI for each cell was computed via a vector averaging method (Swindale, 1998):

$$\text{gOSI }=\text{ sqrt}((\sum {\text{R}}_{\text{i}}*\text{ sin2}\theta_{\text{i}}{}^{\text{2}})\;+\;(\sum {\text{R}}_{\text{i}}*\text{ cos2}\theta_{\text{i}}{}^{\text{2}}))/\sum {\text{R}}_{\text{i}}$$

where *i* represents the *i*th direction (equivalent to 1 − circular variance).

## Results

We made a single adeno-associated virus (AAV; Fig. 1*A*) that contains the genes for jGCaMP8s (Zhang et al., 2021) and ChrimsonR (Klapoetke et al., 2014), separated by the P2A cleavage site. This bicistronic expression strategy allows for the reliable co-expression of both proteins in different cells (Tang et al., 2009). The Cre dependence is provided by the DIO (FLEX) strategy (Schnütgen et al., 2003; Cardin et al., 2009), such that without recombination, the genes are in the antisense orientation to limit leaky expression in the absence of Cre.

### Robust expression of jGCaMP8s and stChrimsonR using a bicistronic construct

For all experiments, we injected the virus into primary visual cortex (V1) of adult *Emx1-Cre* mice, to yield expression in excitatory neurons (Fig. 1*B*). We implanted optical windows over V1 for imaging.

At three weeks postinjection, strong GCaMP fluorescence was visible using fluorescence imaging of the cortical surface through the optical window (*N* = 6 animals injected and measured 22–53 d postinjection; Fig. 1*C*; Extended Data Fig. 1-1). To assess expression in individual cells, we used *in vivo* two-photon calcium imaging. We found robust expression in many cells across multiple imaging depths (Fig. 1*D*). To assess imaging crosstalk, where the imaging laser might in principle activate the opsin, we measured average cell activity and found no sign of crosstalk (2/12 imaging sessions showed significant increases over the 1st 2 s of imaging, *p* < 0.05; 4/12 showed significant decreases, *p* < 0.05; Mann–Whitney *U* test of cell fluorescence between 1st second and 2nd second after imaging onset, Bonferroni correction for multiple comparisons; see Extended Data Fig. 1-2 for time courses).

We find that many or all neurons show GCaMP fluorescence throughout the cell, unlike what is seen with expression of GCaMP alone under control of a single promoter, where GCaMP is often excluded from the nucleus (Tian et al., 2009; T.W. Chen et al., 2013; Packer et al., 2015). Because this might imply some differences in GCaMP trafficking for this bicistronic virus compared with GCaMP expressed with a single promoter, we next compare responses of neurons transfected with this bicistronic virus versus those from neurons transfected with two single viruses that express each of the two proteins (jGCaMP8s and stChrimsonR) separately.

### *In vivo* recording of visually-evoked and spontaneous activity of V1 cells expressing jGCaMP8s-P2A-stChrimsonR

In order to determine whether cells expressing jGCaMP8s-P2A-stChrimsonR exhibit physiologically expected sensory-evoked responses (see Kondo and Ohki, 2016; Zhang et al., 2021; Bounds et al., 2022), we used two-photon imaging to record jGCaMP8s activity from excitatory cells in layer 2/3 of mouse V1 during and between presentations of drifting grating [Gabor patches, 15° full-width half-max (FWHM)] stimuli. We presented gratings across eight orientations (*N* = 20 repetitions per orientation) in random order and computed trial-average ΔF/F_0_ for different orientations. We found strong and widespread visually-evoked activity across the field of view (Fig. 2*A*,*B*). To assess visual selectivity and responsiveness of cells, we calculated single-cell ΔF/F_0_ activity traces across visual presentations and selectivity indices for grating direction (DSI) and orientation (OSI and global OSI, or gOSI; Fig. 2*C* shows selectivity in four example cells and Fig. 2*D* displays activity in 20 example cells across three consecutive trials from one animal). We found many cells respond to drifting grating stimuli (913/1046 cells responsive across *N* = 3 animals, two-sample *t* test, stimulus vs baseline, all stimulus directions pooled; *p* < 0.001 threshold; Bonferroni correction across neurons within each experiment). Many neurons also exhibit spontaneous activity (Fig. 2*D*) between grating presentations.

We first compared visual responses to previous reports from other laboratories. We examined tuning for the direction and orientation of the stimuli and found the distribution of neurons’ tuning was very similar to what has been previously reported with GCaMP6 or GCaMP7 (Kondo and Ohki, 2016; Bounds et al., 2022; and see Niell and Stryker, 2008 for a comparison to electrode recordings). For every cell, in addition to selectivity indices, we also measured preferred direction and orientation and overall responsivity (average activity across tuning curve, see Materials and Methods for details; Fig. 2*G*, green curves). We found the distributions of these tuning metrics align closely with prior reports using calcium indicators (GCaMP6s, Kondo and Ohki, 2016; see their Figs. 3D, 4D and supp Fig. 7D; GCaMP7s, Bounds et al., 2022; see their Fig. 2E). We find that preferred orientations and directions are evenly distributed (Fig. 2*G*) and tuning index distributions are similar to what has been previously reported. In these reports, mean DSI was 0.27 (Kondo and Ohki, 2016), while our mean DSI was 0.21 ± 0.21 (1 SD) Similarly, their mean OSI was 0.62 (Kondo and Ohki, 2016) and 0.56 (Ai203; Bounds et al., 2022), while we report a mean OSI of 0.46 ± 0.28. For gOSI, they report a mean of 0.46 (Kondo and Ohki, 2016), while we report a mean gOSI of 0.31 ± 0.18.

Next, we examined how the bicistronic construct’s visual responses compare to visual responses in data we obtained from transfection with two different viruses, each carrying jGCaMP8s or stChrimsonR (560/643 cells responsive across *N* = 2 animals, two-sample *t* test, stimulus vs baseline, all stimulus directions pooled; *p* < 0.001 threshold; test done with Bonferroni correction across neurons within each experiment). Across all measures of selectivity, stimulus preference, and responsivity, we found that distributions of cell metrics align closely (Fig. 2*E*). These data demonstrate that expression of jGCaMP8s-P2A-stChrimsonR allows for reliable two-photon measurements of sensory-evoked and ongoing calcium activity.

### Stability of visual responses over time in V1 cells expressing jGCaMP8s-P2A-stChrimsonR

A challenging aspect of long-term all-optical experiments is maintaining satisfactory levels of expression over weeks to months. To assess the suitability of our construct for longer-term experiments, we next measured sensory-evoked activity in the same FOV of multiple animals a month apart. Again, we found that V1 cells expressing jGCaMP8s-P2A-stChrimsonR produce robust visually-evoked responses to drifting grating stimuli (Fig. 3*A*,*B*). Using tuning selectivity metrics calculated from responses to gratings of eight different directions (DSI, OSI, and gOSI), as well as a measure of overall responsivity (average across tuning curve activity, see Materials and Methods for details), we found no substantial change in cell responses from two animals a month apart in the same FOV (Fig. 3*C*,*D*). These results indicate that measures of activity from cells expressing our construct are reliably maintained over the span of several weeks, enabling longer-term experiments.

### Holographic pattern stimulation *in vivo* of cells expressing jGCaMP8s and stChrimsonR

One goal of achieving co-expression of an indicator and an opsin is to facilitate all-optical interrogation of brain circuits. Therefore, to test the ability of cells expressing the construct to respond to optogenetic stimulation, we measured jGCaMP8s activity via two-photon imaging *in vivo* (920-nm imaging wavelength, 15- to 20-mW imaging power, 80-MHz pulse rate, 0.19–0.25 nJ/pulse) while stimulating cells with holographic light patterns (1030 nm, 500-kHz pulse rate, 13–16 nJ/pulse). We used holography to simultaneously stimulate cells in mouse V1 with 10-µm diameter light spots in a single depth plane. This stimulation (20 targets, 2.0 mW/target for 300 ms, see Materials and Methods for details; Fig. 4*A*) led to clear stimulation-evoked responses in targeted cells (Fig. 4*B–D*).

We compared the time course of stimulation responses produced with this bicistronic virus to those from neurons transfected with stChrimsonR and jGCaMP8s using two different viruses. We found decay times were similar for the two methods (Extended Data Fig. 4-1), suggesting jGCaMP8s was functioning similarly for both viral expression strategies. In fact, the variability in decay times was smaller with this bicistronic virus (Extended Data Fig. 4-1*C*), perhaps because of lower expression variability of the two proteins with this virus compared with a two-virus approach.

We observed reliable responses to stimulation in a large majority of targeted cells and a few nearby, unstimulated cells (Fig. 4*A–D*, 19/20 targeted cells and 5/69 unstimulated cells above a 7.5% ΔF/F_0_ threshold; see Extended Data Fig. 4-2 for full distribution of responses; Materials and Methods). These reliable stimulation responses were seen consistently across animals (*N* = 3 mice, *N* = 10 or 12 targeted cells, 30/32 total targeted cells and 35/1037 unstimulated cells show responses above a 7.5% ΔF/F_0_ threshold; Extended Data Figs. 4-3, 4-4).

To understand the extent to which the stimulation pattern was precisely exciting the targeted cells and not adjacent cells, we computed the average response around each targeted cell (Fig. 4*E*). We found that the 10-µm excitation light disks we used produced very localized activation (full width at half-max, 13.5 µm estimated via Gaussian fit; Fig. 4*H*). Given we observed responses in a fraction of nearby unstimulated cells, we further compared the photostimulation response in unstimulated cells as a function of distance to the nearest stimulated cell. We find that only cells nearby to stimulated cells (within 40 µm) show a positive response to stimulation, with no responses seen at greater distances (Extended Data Fig. 4-4). These results are in agreement with evidence that cell-specific stimulation drives localized activity in nearby cells within a distance of <40 µm (Packer et al., 2015; Oldenburg et al., 2022).

The holographic approach allows stimulation of many cells simultaneously, and we checked the effects of stimulation at larger numbers of neurons. This stimulation (50 targets, 2.5 mW/target for 150 ms; Fig. 4*I–L*) also produced consistent activation. We found 33/50 targeted cells showed reliable activation (7.5% ΔF/F_0_ threshold, although some cells are activated more strongly than others, Fig. 4*L*; Extended Data Fig. 4-3). When giving input to larger numbers of neurons (e.g., 50), it might be expected that response reliability would be lower than when driving smaller numbers of neurons (e.g., 20, Fig. 4*A–D*) because of recurrent network effects when some stimulated neurons affect other nearby cells (Marshel et al., 2019; Dalgleish et al., 2020; O’Rawe et al., 2022; Oldenburg et al., 2022). We also find more off-target activation in nearby cells when using 50 stimulation targets (45/298 unstimulated cells above a 7.5% ΔF/F_0_ threshold, Extended Data Fig. 4-2 for full distributions, Extended Data Fig. 4-4 for distance-dependent effects), when compared with 20 stimulation targets (Fig. 4*C*). It seems likely that this increase in off-target activation is also because of network effects via postsynaptic summation. In sum, we find that a majority of jGCaMP8s-expressing cells selected for holographic targeting show consistent responses to repeated photostimulation, indicating these cells are successfully co-expressing both indicator and opsin.

## Discussion

In order to facilitate two-photon holographic photostimulation experiments with simultaneous calcium imaging, we designed a Cre-based, single viral approach to express both opsin and indicator in Cre-expressing cells of interest. The single viral approach simplifies experimental preparation by reducing variation because of relative concentrations of opsin and indicator. Our results indicate that cells expressing AAV9-hSyn-DIO-jGCaMP8s-P2A-stChrimsonR exhibit strong and consistent responses to targeted holographic photostimulation. Also, visual responses measured using this approach have population distributions similar to those previously reported with expression of calcium sensors, indicating normal visual function is maintained and neural activity can be reliably recorded.

### Robust activation of neurons with ChrimsonR likely reflects changes in firing rates, instead of precisely timed single spikes

A central appeal of two-photon holography is the ability to dynamically stimulate different groups of cells based on functional properties. However, a limiting factor for such experiments is achieving reliable co-expression of opsin and indicator in many cells throughout the tissue. Recent studies using a variety of opsins and genetic approaches typically find many, but not all cells are responsive to photostimulation (for example, ∼80% with Ai203 transgenic line, Bounds et al., 2022; ∼90% with ChRmine, ∼85% with ChroME2s, ∼75% ChroME2f, Sridharan et al., 2022). On par with prior reports for other opsins, we found similar or greater levels of photoactivatable cells in our experiments (∼80% of cells photoactivatable; Fig. 4; Extended Data Figs. 4-2, 4-3, 4-4). However, our data differ from previous reports with ChrimsonR, which suggest very few ChrimsonR cells are reliably activated with two-photon stimulation (∼10%, see Sridharan et al., 2022, their Fig. 7C,D). This report differs from ours in that they aimed to induce precisely-timed single spikes, and thus used much shorter stimulation periods (5 ms, repeated five times at 30 Hz). Also supporting the idea that stChrimsonR can be used to reliably activate cells, Mardinly et al. (2018) found ∼60% of stChrimsonR-expressing neurons were reliably photoactivated (5-ms stimulation at 0.4 mW/µm^2^, 1040 nm, 2-MHz pulse rate; however, pulse rate differences with our 500 kHz make direct power comparisons difficult). Thus, while ChrimsonR activation produces relatively smaller photocurrents than the ChroME and ChRmine opsins, our work, as well as past work, suggests that stChrimsonR can indeed be used to reliably activate cells *in vivo*.

It could be possible, in fact, that smaller stimulation currents, which modulate the firing rate of neurons without reliably driving single spikes with precise timing, are similar to inputs neurons receive during many forms of visual sensory stimulation. During flashed or drifting grating stimuli (Niell and Stryker, 2008; Busse et al., 2011; Glickfeld et al., 2013), mouse neurons change their firing rates by only up to tens of spikes per second or less, and fire with irregular, Poisson-like timing. Electrophysiological experiments show that activation of stChrimsonR in the visual cortex with longer pulses produces an elevation of neurons’ firing rates while neurons’ firing remains irregular, as occurs with sensory stimuli (O’Rawe et al., 2022). The elevated firing rate continues for as long as cells are illuminated (up to 1000 ms).

In sum, the difference between past low fractions of activated cells with ChrimsonR and the large fractions of excitable cells we show here may be in part because of a difference in stimulation duration and thus total current injected. Longer pulses should be expected to produce more spikes on average and improve the chances of detecting photoactivation, enabling flexible targeting of behaviorally-relevant neurons. One final consideration is that the calcium indicator we used may improve detection. By pairing ChrimsonR with jGCaMP8s, an indicator optimized for the detection of single spikes (with the trade-off of becoming nonlinear with fewer spikes than jGCaMP8m or 8f; see Zhang et al., 2021, their Supp Fig. 6), we improve our ability to detect photoactivation, while prior reports have paired ChrimsonR with the less-sensitive GCaMP6 or GCaMP7 variants.

### Cell responses to both optogenetic stimulation and visual stimulation are robust and stable over time

Many or all neurons transfected with our virus show GCaMP fluorescence throughout the cell instead of being excluded from the nucleus. In single-promoter GCaMP expression, cell-filling with GCaMP is often associated with a pathologic state which develops over time and leads to bright fluorescence no longer modulated by activity (Tian et al., 2009; T.W. Chen et al., 2013; Packer et al., 2015). In this study, however, most or all neurons are filled, but the filled neurons are neither extremely bright, nor static. In fact, our two-photon stimulation results (Fig. 4) and our visual stimulation results (Fig. 2) suggest that the filled neurons are responding to input and reflecting these changes in GCaMP fluorescence, and that our ability to measure responses is maintained over weeks (Fig. 3). Our observations of cell responses to visual stimulation (Fig. 2*E*) and photostimulation (Extended Data Fig. 4-1) also match well with responses measured in animals expressing jGCaMP8s and stChrimsonR via separate viruses. Visual responses across the population from the different preparations are highly similar (Fig. 2*E*), indicating many of the P2A-expressing cells respond as expected despite apparent cell-filling. Further, Tian et al. (2009) reports that unhealthy nuclear-filling leads to an increased signal decay time, which we do not observe between the P2A and dual-virus preparations, suggesting cells expressing our P2A virus remain healthy. In fact, we find that cells expressing our P2A virus show slightly shorter decay times as compared with cells in the dual-virus preparation, and also exhibit less variance in decay times (Extended Data Fig. 4-1), perhaps because of less variation in relative levels of expression between opsin and indicator. The filling might arise because P2A cleavage can result in several added amino acids from the P2A linker that may yield a GCaMP protein trafficked slightly differently than the GCaMP constructs expressed with a single promoter (Kim et al., 2011). However, the neural responses we measured with this virus were robust, stable, and were similar to sensory-evoked responses previously measured in other work (Fig. 2). Therefore, if there is some difference in protein trafficking, it seems to leave the GCaMP responses to neural activity intact.

### Future and conclusion

Currently, most studies employing all-optical methods for circuit dissection have focused on cortical areas. However, holographic stimulation can be used to investigate network function in other brain regions, as in the olfactory bulb (Gill et al., 2020) or the basolateral amygdala (Piantadosi et al., 2022). While viral tropisms may affect whether any given virus that works well in the cortex also works well in other brain regions, bicistronic delivery methods of both opsin and indicator will simplify testing of expression strategies.

In the present work, we build on previous efforts using separate viruses to express various GCaMP indicators (GCaMP6f, GCaMP6s, jGCaMP7s) with ChrimsonR (in mice, Attinger et al., 2017; Stamatakis et al., 2018; Chettih and Harvey, 2019; Gill et al., 2020; Daie et al., 2021; Akitake et al., 2022; O’Rawe et al., 2022; and in zebrafish, Förster et al., 2017) and on other efforts using linking peptides to simultaneously express both opsin and indicator (GCaMP6m-P2A-ChRmine, Marshel et al., 2019). Our bicistronic virus adds to the growing genetic toolbox enabling future experiments requiring flexible patterned stimulation methods by offering a single viral approach to express the latest GCaMP8s indicator alongside the ChrimsonR opsin in cells of interest.

## Synthesis

Reviewing Editor: David Schoppik, New York University Decisions are customarily a result of the Reviewing Editor and the peer reviewers coming together and discussing their recommendations until a consensus is reached. When revisions are invited, a fact-based synthesis statement explaining their decision and outlining what is needed to prepare a revision will be listed below. The following reviewer(s) agreed to reveal their identity: Eirini Papagiakoumou.

After discussion, we felt that there were two useful points to consider for your revision:

R1 felt that “The new crosstalk figure (extended figure 1-2) claims that if crosstalk is present, fluorescence should increase over the first several seconds of imaging. Couldn’t crosstalk occur so quickly that there is no build up of higher activation compared to imaging onset? Couldn’t crosstalk be significant but slight enough that with a mere second or of baseline data you cannot detect a difference? I am not aware of any studies showing this method of quantifying crosstalk combined with ground truth spiking data to ensure that it is accurate. As mentioned above, I think the manuscript is stronger without this data.”

R2 disagreed: “I believe this [the crosstalk figure] remains useful, as I couldn’t find any work characterizing the crosstalk with ChrimsonR. There is a nice characterization in Sridharan et al. Neuron 2022 for ChroME and ChRmine, but not for Chrimson, and even more not with GCaMP8. I agree that ideally such a study would be better done with electrophysiology, nevertheless I don’t believe that crosstalk Calcium rise would be so fast, that we could not see it, especially by scanning at so low power (they use 15-20 mW for Calcium imaging, the lowest power I’ve ever seen in such a paper), and with ChrimsonR, that is not the most efficient opsin. We all know that scanning with a laser beam for sure does something to the state of the neurons when they express an opsin. But the question is if the depolarization is enough to induce activity. And if this activity is not detectable by the Calcium sensor in an all-optical experiment, then this is already useful information. This of course needs always to be accordingly interpreted in the framework of each specific study. Besides, we are talking about a supplementary figure here, that doesn’t harm!”

In the interest of time I’ve decided to just share the Reviewers’ thoughts about the figures and let you decide; I’m personally with R2 that a supplemental figure never hurt anyone so feel free to keep it.

One last point: For the figures on off target effects (new Fig 4 panels, extended data fig 4-2), the visualization of the heatmap away from the mean cell is not useful. Given an approximately random distribution of off target cells around a cell it is easy for off targets to average out. A more useful visualization would be to show a scatter plot of the DFF response of unstimulated cells on the y axis and distance from the nearest target on the x axis, or something similar.
