# Supplementary figures and images for: Bicistronic Expression of a High-Performance Calcium Indicator and Opsin for All-Optical Stimulation and Imaging at Cellular Resolution
Source: eNeuro. 2023 Mar 28;10(3):ENEURO.0378-22.2023. doi: 10.1523/ENEURO.0378-22.2023 (PMC10062490; doi:10.1523/ENEURO.0378-22.2023)

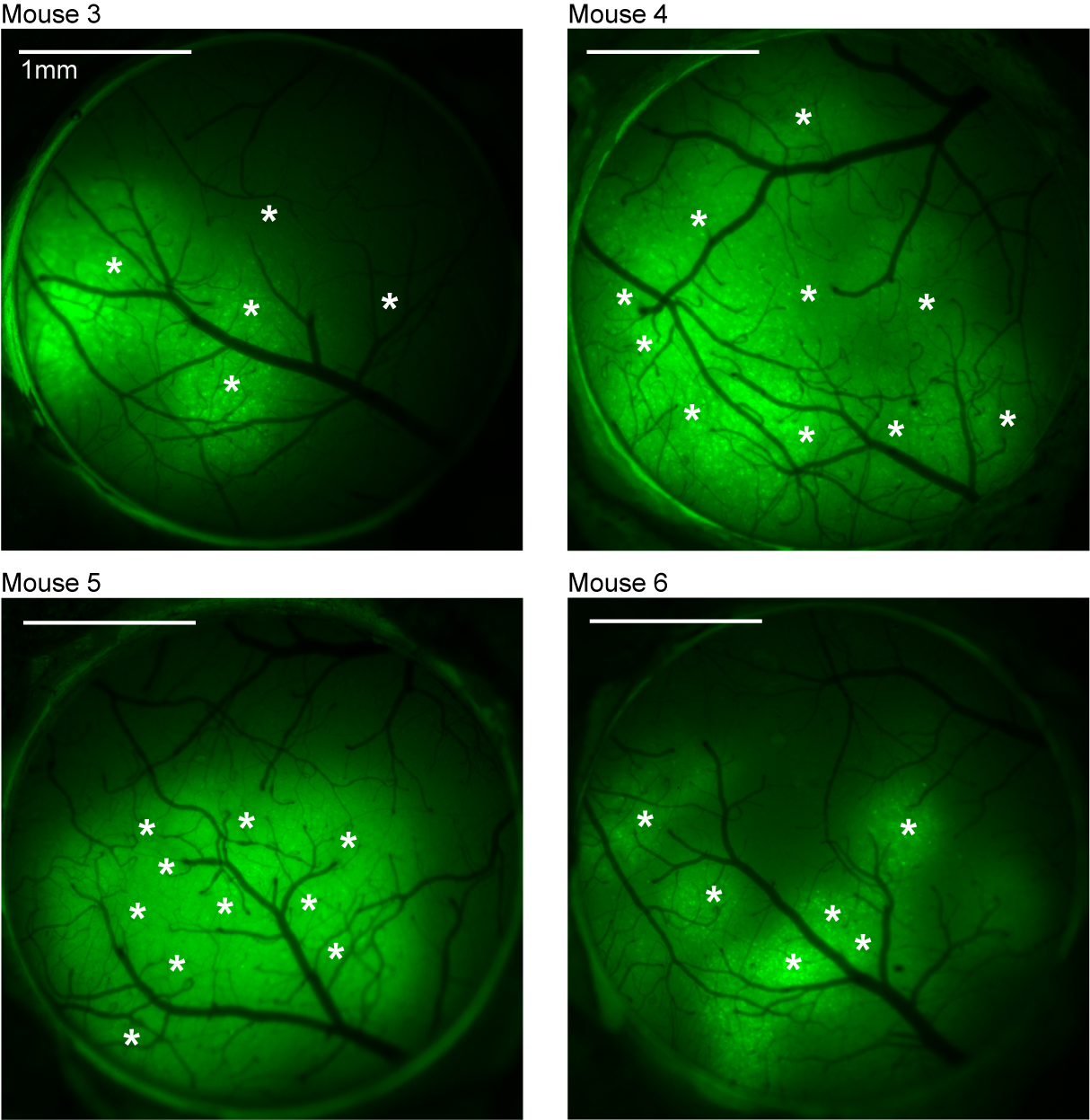

Supplement: Extended Data Figure 1-1 — Widefield fluorescence imaging of jGCaMP8s-P2A-stChrimsonR expression. Widefield fluorescence imaging through 3-mm optical windows in experimental mice. White stars: injection sites for each mouse (mouse 3: N = 5 sites, mouse 4: N = 10 sites, mouse 5: N = 10 sites, mouse 6: N = 6 sites). Images were acquired 22–53 d after injection. Download Figure 1-1, TIFF file. [file enu-eN-MNT-0378-22-s02.tif]

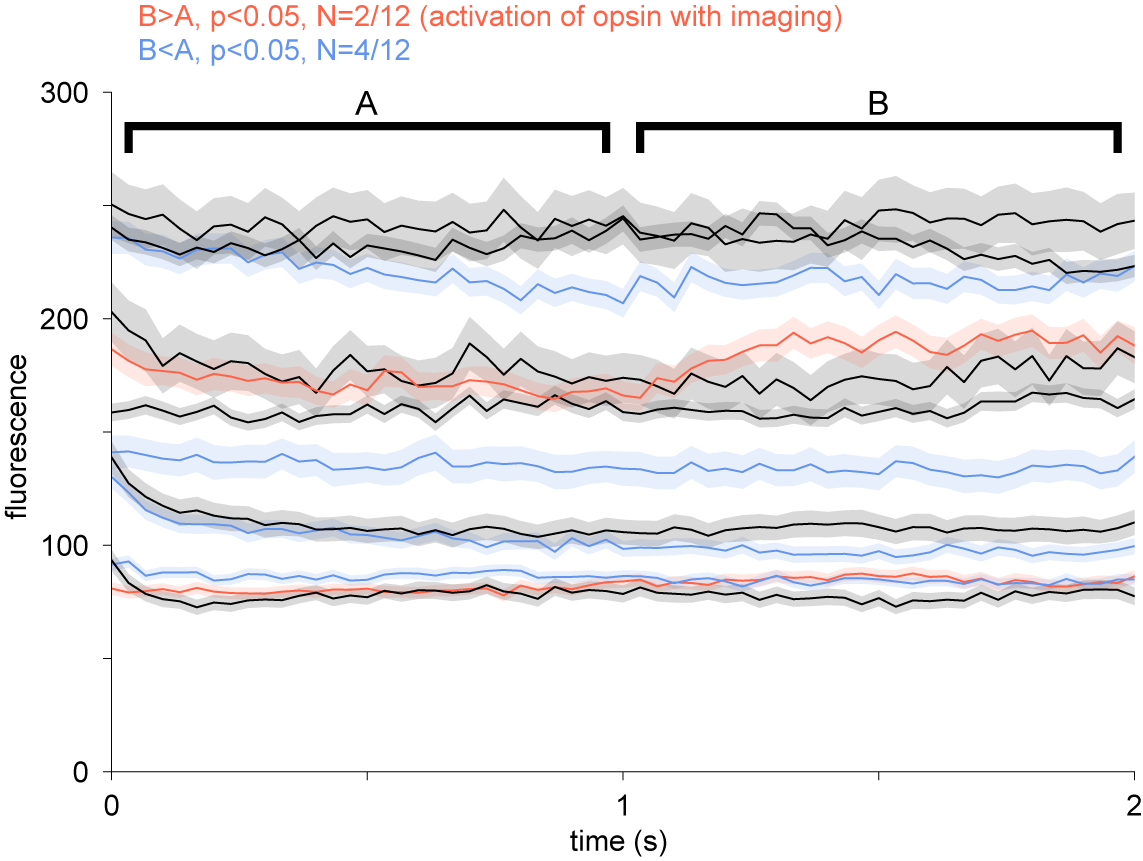

Supplement: Extended Data Figure 1-2 — Minimal signs of cross talk activation from resonant-galvo scanning at imaging onset. Imaging cross talk should lead to elevated fluorescence at onset of imaging. Cell-averaged fluorescent traces in imaging FOVs (N = 12 FOVs from N = 5 mice) across first 2 s of imaging do not show signs of widespread cross talk in activation from the imaging laser (920 nm). Only a few sessions (2/12) show small increases in fluorescence over the first few seconds after imaging onset, as would be expected if imaging cross talk were occurring (significantly higher levels of fluorescence in the 2nd second of imaging vs the 1st second p < 0.05, Mann–Whitney U test with Bonferonni correction for multiple comparisons). On the other hand, however, four of 12 imaging sessions show significantly lower levels of fluorescence, arguing against cross talk activation (p < 0.05, Mann–Whitney U test with Bonferroni correction for multiple comparisons). Together, this suggests that elevated fluorescence at the start of imaging, due to cross talk, is not a concern with this preparation. Download Figure 1-2, TIFF file. [file enu-eN-MNT-0378-22-s03.tif]

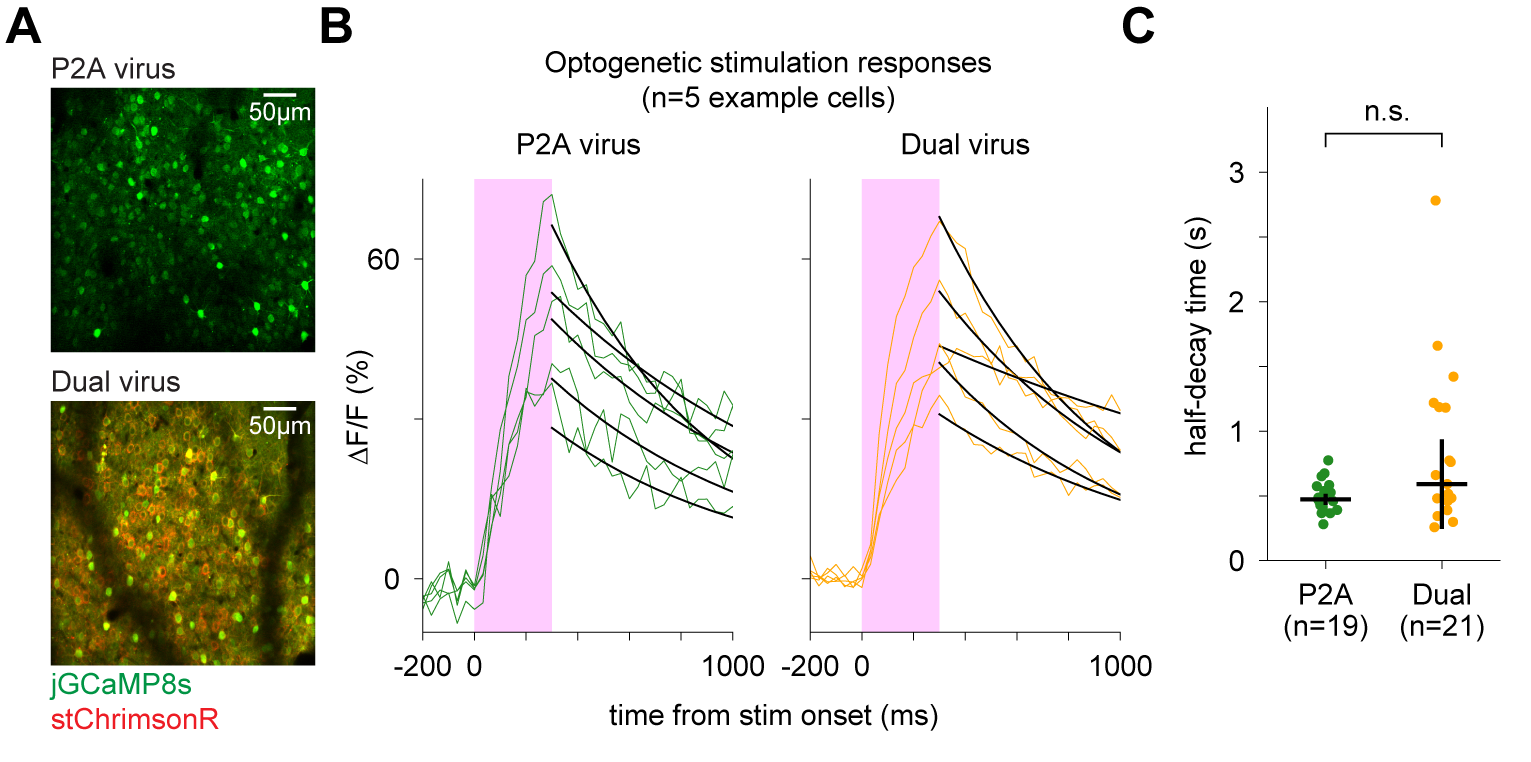

Supplement: Extended Data Figure 4-1 — GCaMP decay times to photostimulation responses are similar between P2A and dual-virus preparations. A, Two-photon imaging FOV (414 × 414 µm) in layer 2/3 of mouse V1 for both a P2A mouse (top) and a dual-virus mouse (bottom). Imaging collected at 30-Hz frame rate, 10-mW power, 920 nm. B, Trial-average photostimulation responses in five example cells from a P2A (left, green lines) or dual-virus (right, orange lines) mouse. Black lines, Exponential decay functions fit to the 600-ms period following stimulation offset for all photostimulated cells to estimate decay times (Materials and Methods). Stimulation power was 2.0 mW/target (P2A) or 2.5 mW/target (dual-virus) for 300 ms using 10-µm diameter disk patterns. C, Half-decay times for photostimulated cells from both mice. Horizontal black lines, Mean half-decay time amongst cells. Vertical black lines, SEM. Half-decay times are not significantly different between P2A-expressing and dual-virus-expressing cells (p = 0.37, two-sample t test). This figure: N = 2 mice. A–C, Mouse 1 and a dual-virus mouse. Download Figure 4-1, TIFF file. [file enu-eN-MNT-0378-22-s04.tif]

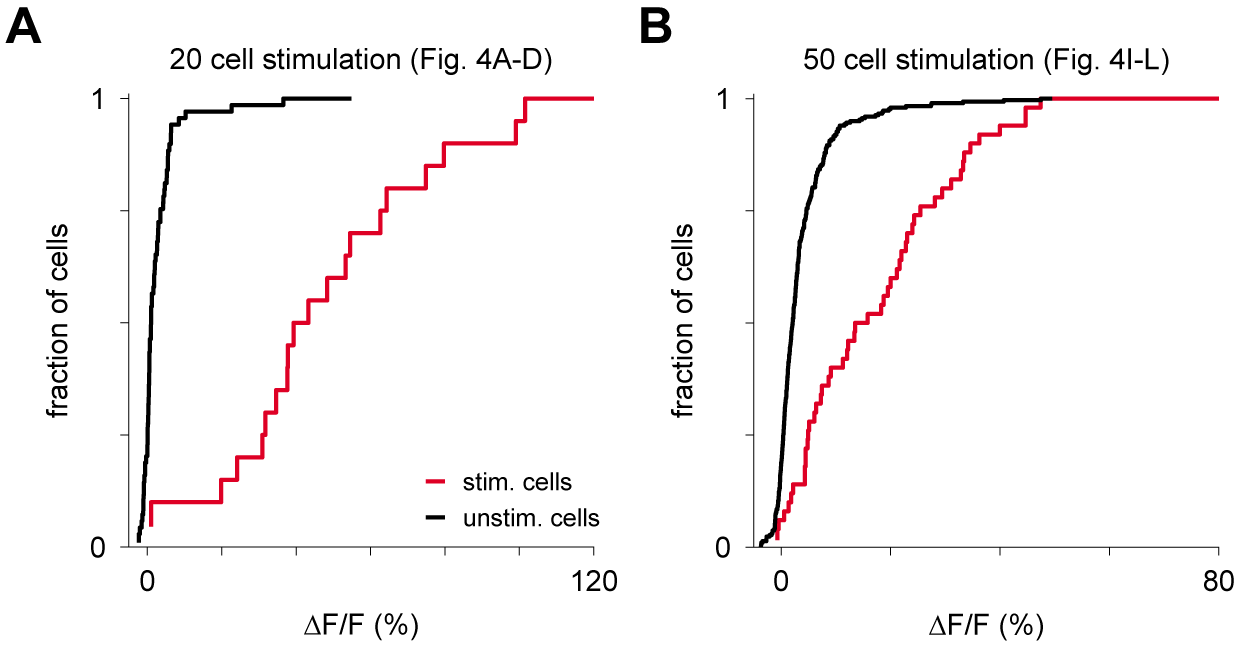

Supplement: Extended Data Figure 4-2 — Distribution of responses to photostimulation in stimulated and unstimulated populations. A, Cumulative distribution functions of photostimulation responses (from data in Fig. 4A–D) averaged over a 1-s period following stimulus onset in both stimulated cells (red) and unstimulated cells in the FOV (black). Using a 7.5% ΔF/F0 threshold, we found 19/20 stimulated cells and 5/69 unstimulated cells show reliable activation. B, Same as in A, but for data from Figure 4I-L. Using a 7.5% ΔF/F0 threshold, we found 33/50 stimulated cells and 45/298 unstimulated cells show reliable activation. To account for concerns of stronger neuropil contamination in response calculations when stimulating larger groups of cells, we also used a ring-based neuropil correction via Suite2p and found minimal differences in responses (35/50 stimulated cells and 41/298 unstimulated cells above a 7.5% ΔF/F0 threshold), implying neuropil signal was not contaminating cell responses. This figure: N = 1 mouse. A, B, Mouse 1. Download Figure 4-2, TIFF file. [file enu-eN-MNT-0378-22-s05.tif]

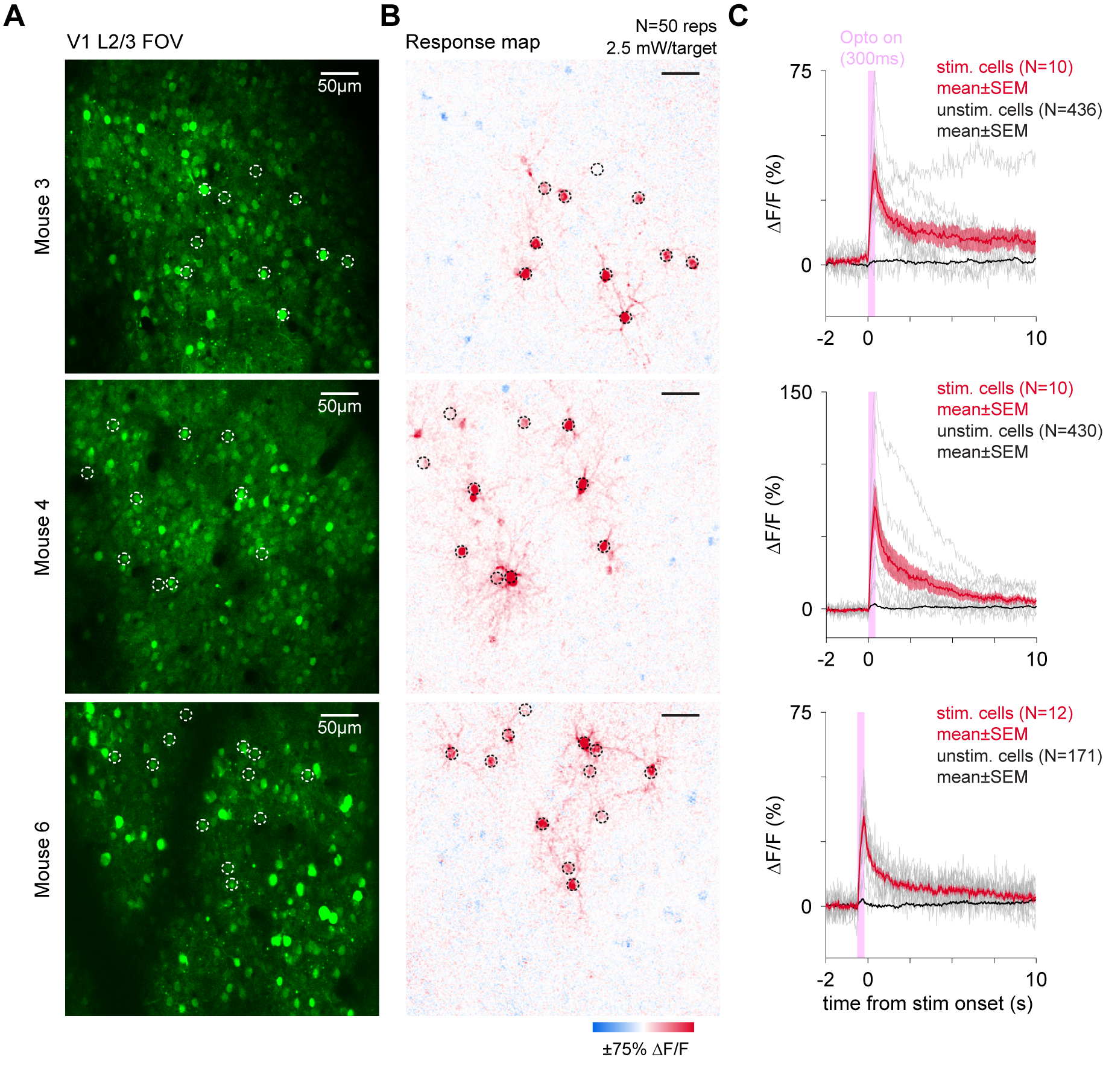

Supplement: Extended Data Figure 4-3 — Holographic stimulation of cells expressing jGCaMP8s-P2A-stChrimsonR. A, Two-photon imaging FOV (414 × 414 µm) in mouse V1 in three example mice. [Depths: 130 µm (top), 140 µm (middle), and 110 µm (bottom); each in L2/3, depth differences do not produce systematically different responses.] Imaging at 30-Hz frame rate, 15-mW imaging power, 920 nm. White dashed rings, Holographic stimulation pattern (top: N = 10 targets, middle: N = 12 targets, bottom: N = 10 targets). B, Corresponding stimulation response maps showing mean optogenetically-evoked activity (ΔF/F0; N = 50 stim reps). Stimulation power 2.5 mW/target, stim duration 300 ms, 10-µm diameter disk patterns. C, Trial-average activity of all stimulated cells (light gray lines) plotted with mean ± SEM activity across all stimulated cells (red lines) and all unstimulated cells (black lines). SEM is present for unstimulated cells, but small relative to the size of the plotted line. Fraction of responsive stimulated cells: (top) 9/10, (middle) 11/12, (bottom) 10/10 (using 7.5% ΔF/F0 threshold, see Materials and Methods for details). Fraction of responsive unstimulated cells: (top) 11/436, (middle) 19/430, (bottom) 5/171. Download Figure 4-3, TIFF file. [file enu-eN-MNT-0378-22-s06.tif]

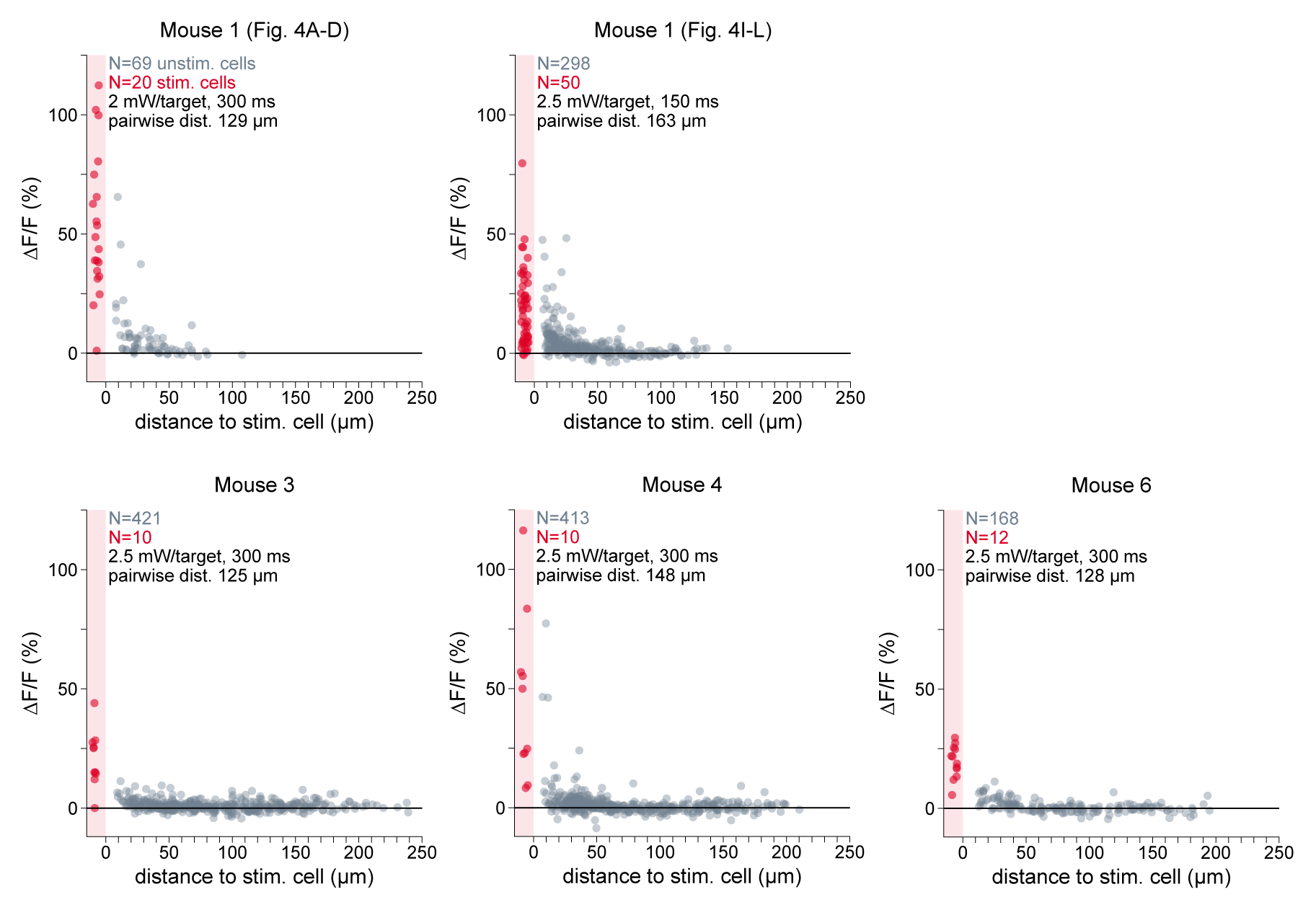

Supplement: Extended Data Figure 4-4 — Spatial extent of photostimulation effects in unstimulated cells. Trial-average responses to photostimulation. Red points, Stimulated cells. Gray points, Unstimulated cells. Unstimulated cells are plotted as a function of distance to the nearest stimulated cell in the FOV. Stimulation power, duration, and the mean pairwise distance between stimulated targets is shown for all photostimulation experiments in P2A animals. This figure: N = 4 mice. Download Figure 4-4, TIFF file. [file enu-eN-MNT-0378-22-s07.tif]
